# Prognostic Power of Foot Mobility in Identifying the Risk of Musculoskeletal Injuries: A Cross-Sectional Study of Male Volleyball Players at Different Competitive Levels

**DOI:** 10.3390/jcm13051189

**Published:** 2024-02-20

**Authors:** Jarosław Domaradzki, Dawid Koźlenia, Marek Popowczak, Jaromir Šimonek, Ľubomír Paška, Pavol Horička

**Affiliations:** 1 Faculty of Physical Education and Sport, Wroclaw University of Health and Sport Sciences, I.J. Paderewskiego 35, 51-612 Wroclaw, Poland; 2 Department of Physical Education and Sport, Constantine the Philosopher University in Nitra, Tr. A. Hlinku 1, 94901 Nitra, Slovakia; jsimonek@ukf.sk (J.Š.); lpaska@ukf.sk (Ľ.P.); phoricka@ukf.sk (P.H.)

**Keywords:** volleyball players, top level, academic level, navicular drop test, injuries, lower extremity, ROC curve, injury risk factors

## Abstract

(1) Background: The arch structure and mobility of the foot are considered injury risk factors in volleyball. However, there are limited studies presenting differences in injury prevalence and the risk of lower limb injuries in relation to the competitive level in male volleyball. Therefore, the main aim of the current study was to evaluate foot mobility (through navicular drop test) as an injury risk factor in volleyball players from different competitive levels. (2) Methods: The reliability and usefulness of navicular drop testing were initially assessed in test–retest procedures (based on a sample of eight participants and 16 feet measurements), with primary analyses conducted using foot measurements of the twelve top-level volleyball players (24 feet) and eighteen academic-level volleyball players (36 feet). The modified navicular drop test was conducted, and the feet were classified based on arch height, and injury prevalence was retrospectively assessed with a previously validated questionnaire. Chi-squared tests, receiver operating curves, and logistic regression were used as statistical methods. The navicular drop test was verified as a reliable tool by intraclass correlation coefficient (ICC) (3.1) analysis. (3) Results: There were no significant differences in injury prevalence between academic- and top-level volleyball players, though there was a significant relationship between pronated foot and injury risk independent of competitive level. Generally, for both groups, thresholds above 10 mm of the navicular drop were predictors of lower limb injuries. The risk of injury if the foot was pronated ranged from 70% (academic level) to over 90% (top-level players). However, no statistically significant effect of competitive level on the chance of injury was observed. (4) Conclusions: Our study found a high prevalence of foot injuries independently of competitive level. There was a relationship between pronation of the foot and the risk of injury. However, the risk of lower limb injury was higher in pronated top-level players. Also, a navicular drop greater than 10 mm was an excellent predictor of injuries at both competitive levels.

## 1. Introduction

Foot structure, particularly the medial longitudinal arch (MLA), is considered an important factor determining shock absorbance and shake energy reduction during walking, running, and jumping [1,2]. However, arch function is determined by foot bony structure, ligament stability, and muscular performance [3,4].

Low-arched (LA) foot, commonly known as flat or planus foot, is related to pronation, while high-arched (HA) foot is related to supination. Both LA foot and HA foot are perceived to be risk factors for injury in many sports activities, especially running and team games [5,6,7]. Evidence from studies confirmed a higher risk of injury in players with a pronated or supinated foot compared with a neutral foot. A planus or pronated foot loads the medial part of the foot, while a supinated foot loads the lateral structures [8], suggesting different possible injury types. Moreover, medial and lateral loads may be transferred up the entire lower extremity and coincide with knee structure and function, which may increase the risk of injury [8].

Foot structure determines mobility, and LA individuals usually have more mobile feet than HA individuals [5]. Classically, mobility can be tested using palpation or with electronic sensors. A palpation method, the navicular drop test (NDT) developed by Brody [9], is still used, particularly in field examinations. This simple but effective method was validated many times and found to be reliable and useful for assessing the functions of the foot [10,11]. The test is based on navicular drop assessment, which relies on shifts in the navicular bone between a relaxed, non-weighted position (e.g., sitting) and a weighted position (e.g., standing). LA individuals with more mobile feet require increased control of the foot structures during typical movement, with the control placed on ligaments and tendons, which can result in more injuries to these structures [5].

Injury risk increases if feet are regularly exposed to strong loads related to jumps, landings, pivots, or running. Such situations take place in sports activities, particularly during team games. One of the most common lower extremity injuries is ankle sprain [12,13], which is connected to muscle strains and often observed in volleyball. Despite the lack of physical contact between players, in contrast to other team games, lower limb injuries occur during spiking and blocking through unintentional and illegal intrusion of a player into an opponent’s court [14]. Furthermore, repetitive jumping and unbalanced landings, particularly on one leg, cause overuse and acute injuries [15,16,17]. One-leg landings have been reported to have higher ground reaction forces and muscle activity than two-footed landings [18]. Another common injury type typical of volleyball activity, such as jumping and landing, is knee distortion [19], leading to patellar tendon tendinopathy, subpatellar pain, anterior cruciate ligament (ACL) tears, or meniscus tears.

The risk of lower limb injuries increases if foot posture is poor, with foot pronation considered a potential injury risk factor due to lower extremity overuse [20]. The mechanics are connected to tibial internal rotation via foot and knee joint coupling. Meanwhile, foot supination has been linked to lower extremity injury via increased limb stiffness [21]. However, the risk of injury is related to player technique and experience and may also be linked to competitive level [19].

Epidemiological studies on injuries in professional and elite volleyball players discovered that over 50% suffered from at least one injury during a session [20], with one-quarter of injuries located in the foot (mostly ankle sprains) [21]. Statistics presented by the Fédération Internationale de Volleyball (FIVB) Injury Surveillance System (ISS) indicate a growing tendency in the past ten years (from 2010 to 2020). The study showed that seniors had a higher injury incidence (11.9 per 1000 players) than juniors [19] and were at a two-fold greater risk of injury.

There are limited data on injury risk in collegiate male volleyball players compared with elite, top-level professional players [19], with most evidence gathered from female players. Indeed, studies on injury epidemiology in women exceed those in men, although there are significantly more National Collegiate Athletic Association (NCAA) female volleyball teams than male teams (data from 2022 [19]), and volleyball is more popular among females at universities [22,23]. Nonetheless, a few studies on NCAA men’s volleyball reported an injury rate of 4.69 per 1000 players [24], with acute injuries being more common than overuse injuries (30.5%) and greater during competitions than practice.

The results to date lack a direct comparison of the risk of injuries between top-level professional and academic-level male volleyball players [19,20,21,22,23,24]. Moreover, to the best of our knowledge, there are no comparisons of the prognostic potential of the NDT in detecting the risk of foot musculoskeletal injuries in male volleyball players with the power to distinguish between top-level and amateur players. Therefore, the main aim of the current article was to evaluate foot mobility (using the NDT) as an injury risk factor in male volleyball players at different competitive levels. Specifically, we asked: (1) whether there is a relationship between foot mobility and the prevalence of injuries in top-level and amateur volleyball players, (2) whether the NDT has the power to detect the risk of injury at different competitive levels and whether there are NDT thresholds for increasing risk, and (3) and whether the probability of injury is related to foot mobility.

## 2. Materials and Methods

### 2.1. NDT Measurement Reliability

Before taking the main measurements for this study, their reliability was assessed by one of the authors, a human anatomy teacher trained in practical and functional anatomy and palpations, using a within-day test–retest of the NDT. The first step was to establish the number of participants needed to assess the NDT, which used the formula presented by Walter et al. (1998) [25]. For an intraclass correlation coefficient (ICC) (3.1) model with a minimally acceptable reliability of 0.60, expected reliability of 0.90, alpha-level of 0.05, power (1-beta) of 0.80, two repetitions, and a potential dropout of 10%, it was determined that a minimum 16 measurements were needed.

The intra-rater reliability testing adopted the following strategy: (1) an independent sample of each foot, (2) within-day reliability (successive measurements for each participant), and (3) a modified NDT procedure, as previously described [9,26].

The 18 feet of the nine remaining participants (males, aged 21.1 ± 1.2, body height of 182.5 ± 6.2, body mass of 73.5 ± 6.5, and a body mass index [BMI] of 22.1 ± 2.3) were included in the intra-day comparison of the navicular drop and were measured twice by one rater. BMI was calculated using the formula: BMI = body mass [kg]/body height [m]^2^. A sample of nine healthy adult male volunteers was recruited from university students. The inclusion criteria were the lack of any injury during the six months before the examination, the lack of congenital foot deformity, and the lack of pain in any part of the body upon standing. Each subject underwent a test–retest study workflow, where navicular height (NH) during the stance phase was assessed with two markers on the left and right foot (a description is presented below in the procedure section).

### 2.2. Sample Size for the Main Analysis

Before recruitment, the sample size was determined using receiver operating curve (ROC) analysis. The sample size for the ROC analysis was calculated with the pROC R language library [27] and MedCalc software (MedCalc Version 22.016, MedCalc Software Ltd., Ostend, Belgium, free trial version). Based on 80% total power (1-beta), an alpha level of 0.05, an area under the ROC curve (AUC) of 0.75, a null hypothesis value of 0.5, and a ratio of sample size in the negative|positive groups of 2, this study required 15 positive cases, 30 negative cases, and a total sample size (both groups) of 45. In addition, the sample size for the comparison of the AUC for the two groups was calculated. All the above parameters were calculated using a moderate correlation of 0.3 and a difference in AUC of 0.3, resulting in a minimal number of 48 measurements. However, considering a potential dropout of 20%, the final number of measurements involved in this project was 58. In the current work, feet measurements were treated independently, so 58 feet required 29 individuals.

### 2.3. Participants

Two groups of participants were recruited, with the top-level players including twelve healthy professional male volleyball players (age of 22.5 ± 3.2, body height of 188.4 ± 7.8, and body mass of 84.2 ± 10.6) from the 1st division of the Slovakian National Volleyball League club UKF Nitra. Although the team consisted of 16 players, four were absent during this study. The second group included 18 academic-level amateur male volleyball players (age of 20.1 ± 2.4, body height of 185.8 ± 7.6, and body mass of 79.4 ± 13.3). Of 31 students who declared volleyball practice in the universities’ teams, 6 did not agree to be examined, 4 were excluded due to health problems (with respect to the exclusion criteria), and 3 were not able to take part due to private duties conflicting with the date of examinations. The subject exclusion criteria were (1) injury history shorter than three months before the test, (2) deformity or lower limb surgery, and (3) pain in any part of the body that might affect athletic performance. Participants performed 4–5 training sessions per week with a duration of 90–120, with an additional match. Top-level players’ weekly training volume was 120 ± 21.21 min/week, whereas academic-level players trained 100 ± 11.15 min/week.

The objectives, procedures, and details of this study were explained. The study protocol was approved by the Human Ethics Committee of Wroclaw University of Health and Sport Sciences, Poland (consent No. 16/2018).

### 2.4. Procedure

All measurements were taken at the same time for each participant. Elite volleyball players were measured during evening practice (between 6.30 and 8.00 p.m.), and academic-level (amateur) players were measured between 10.00 and 12.00 a.m. during breaks in academic lectures. The sequence of the measurements was constant.

#### 2.4.1. Anthropometric Measurements

Body height measurements were taken with an accuracy of 0.1 cm using an anthropometer (SECA manufactured, Hamburg, Germany. Quality control number C-2070). Body mass and body fat mass were measured with the InBody230 body composition analyzer (InBody Co. Ltd., Cerritos, CA, USA). Foot length was recorded with a spreading caliper (SECA manufactured, Hamburg, Germany. Quality control number C-2070). The maximal foot length was measured from the back (heel tuber) to the tip of the longest toe (1st or 2nd).

#### 2.4.2. Navicular Drop Test—Modification

The vertical navicular drop measures the difference in NH between a subtalar neutral and weight-bearing position [9], indicating mid-foot mobility, and it is useful as a clinical tool. Vertical shift (movement) in the mid-foot is a meaningful indicator of foot function [28]. The classification of the NDT was based on Brody [26]: supinated foot < 5 mm, neutral foot 5–9 mm, and pronated foot 10–15 mm.

The same researcher who measured the participants for validation carried out all the measurements. The navicular tuberosity was identified and marked with a black hypoallergic marker on the left and right feet of barefoot sitting players (knees were flexed at 90°). The neutral position of the subtalar joint was found with palpation (the head of the talus was palpated on both sides with the thumb and index finger). A blank, stiff paper card was placed on the medial side of the foot, with the navicular tuberosity marked using a ruler. A modification of the original NDT was used in the examinations. The sit-to-stand version of the test was conducted (SSNDT) [26]. Therefore, NH was measured in the standing position with a relaxed bipedal stance, using approximately 80% body mass bearing and the range of vertical ground reaction force at midstance, while the other foot rested on a stool elevated to the same height as the scale [26]. The navicular tuberosity height was marked on the card, and the SSNDT was calculated as the difference between NH at rest and standing. The modified variant of the NDT used is more reliable (ICC = 0.72) than the original variant (ICC = 0.37 to 0.68) [27,29].

#### 2.4.3. Recording Injuries—Injury History Questionnaire

Only musculoskeletal injuries (muscle, tendon, bone, joint, or ligament injuries) were recorded and used to calculate injury prevalence, and only the included foot and knee injuries were analyzed. Injury occurrence was defined based on Widuchowski and Widuchowski (2005) [30] and described as complaints during physical activity resulting in pain and discomfort in the locomotor system, causing temporary limitations or a complete inability to continue physical activity. Injuries in the sample group were collected retrospectively using the Injury History Survey (IHQ), which is a questionnaire about injuries to the locomotor system during physical activity that was presented and validated elsewhere [31]. The IHQ Cronbach’s alpha coefficient was 0.836, indicating high reliability and repeatability of the survey [32].

### 2.5. Statistical Analysis

The Shapiro–Wilk test was used to evaluate the normality of data distribution for the anthropometric measurements and SSNDT. Descriptive statistics were presented as means, 95% confidence intervals of the mean (95%CIs), and standard deviation (SD).

Intra-rater within-day test–retest reliability was evaluated using the ICC (3.1) model (according to Shrout and Fleiss [33]), which corresponds with a two-way mixed effect, absolute agreement, and single-rater reporting standard [34].

The ICC was classified according to Koo and Li as follows [34]:

Less than 0.50: Poor reliability.

Between 0.5 and 0.75: Moderate reliability.

Between 0.75 and 0.9: Good reliability.

Greater than 0.9: Excellent reliability.

The statistical significance of the difference between the two groups was tested using Student’s *t*-test for independent groups. Comparisons of the mean for two factors, competitive levels (academic and top), and type of foot (neutral and pronated) were performed using a two-way analysis of variance (ANOVA) with Tukey’s post hoc test.

The main analysis approach used a sequence of three complementary methods, including chi-squared tests to evaluate the prevalence of the injuries and differences in fractions of injured and non-injured between two groups, ROC curves to study the performance of the SSNDT in detecting the risk of injury and establishing the thresholds, and logistic regression to predict the risk of injury based on the SSNDT and membership in different competitive levels. Such a procedure was used previously in different problems and found to be an effective strategy [35].

In addition, the Youden index was calculated using the formula:

J = maximum (sensitivity + specificity − 1); over all cut–points c; −∞< c < ∞.

The Youden index, based on the ROC curve sensitivity and specificity values, allows for the determination of the optimal cut-off point [36].

A *p*-value < 0.05 was considered statistically significant. The calculations were carried out using Statistica 13.0 (StatSoft Poland 2018, Cracow, Poland).

## 3. Results

The reliability of intra-rater measurements during within-day test–retest was evaluated with the ICC (3.1) model. The descriptive statistics of the sample of nine participants (18 feet) needed for validation are presented in Table 1. Intra-rater reliability calculated for two successive measurements of the within-day SSNDT (SSNDT 1st—first measurement of the day, SSNDT 2nd—second measurement of the day) was placed in a good quality range of classification (ICC = 0.82, 95%CI: 0.55–0.919, F = 9.18, *p* < 0.0001).

The descriptive statistics of the anthropometric and SSNDT measurements for volleyball players (whole group and separated in competitive levels) are presented in Table 2.

There was a significant difference in the age of the players, with the top-level players being older (*p* = 0.005). There were no differences in other anthropometric or foot measurements (*p* > 0.05).

Out of 30 men, 16 (26.7%) had suffered from musculoskeletal injuries. For foot injuries, out of the 24 feet of the top-level players, 6 (25.0%) had experienced at least one injury during the year before the examination. Meanwhile, of the 36 feet of the academic-level players, 10 (27.8%) suffered from injuries. The prevalence of injuries was very similar, and there were no significant differences in the proportions of injured and non-injured between both groups of players (χ^2^ = 0.06, *p* = 0.811, φ = −0.03) (Table 3).

Studying the type of foot showed a lack of supinated feet in both groups and the presence of neutral and pronated feet. Mean values for the academic level were 7.12 mm (neutral feet) and 12.59 mm (pronated feet), and the mean values were 6.67 mm (neutral feet) and 11.33 mm (pronated feet) for the top level. The comparison of foot type (neutral and pronated) showed a statistically significant effect of competition level (F = 5.60, *p* = 0.021) and type of foot (F = 197.37, *p* < 0.001). However, there was no interaction term (F = 1.28, *p* = 0.26), suggesting an additive and combined effect of the factors. Tukey’s test showed significant differences between academic-level players with pronated feet and neutral feet (*p* < 0.001), top-level players with pronated feet and neutral feet (*p* < 0.001), and academic- and top-level players with pronated feet and top-level players with neutral feet, and vice versa (both *p* < 0.001). Non-significant differences were observed between academic- and top-level players with neutral feet (0.800) and pronated feet (0.142).

The analysis of the structure of injuries in relation to the type of foot (neutral or pronated) showed a significant relationship between pronated feet and the prevalence of injuries in the whole group. Seventy-five percent of the pronated feet (*n* = 15) were injured, while only 25% (n = 5) were not (χ^2^ = 0.06, *p* = 0.811, φ = −0.03) (Table 3). There were significant relationships between pronation of the foot and injuries in both groups of players (χ^2^ = 27.6, *p* < 0.001; χ^2^ = 11.2, *p* < 0.001, respectively). However, the relationship was stronger in the academic players (φ = 0.86) than in the top-level players (φ = 0.68) (Table 3).

The next step of the analysis was to assess the diagnostic potential of the SSNDT in predicting the risk of injuries using the AUC. ROC curves for academic and top-level players were drawn, and comparisons were made.

**Figure 1:**
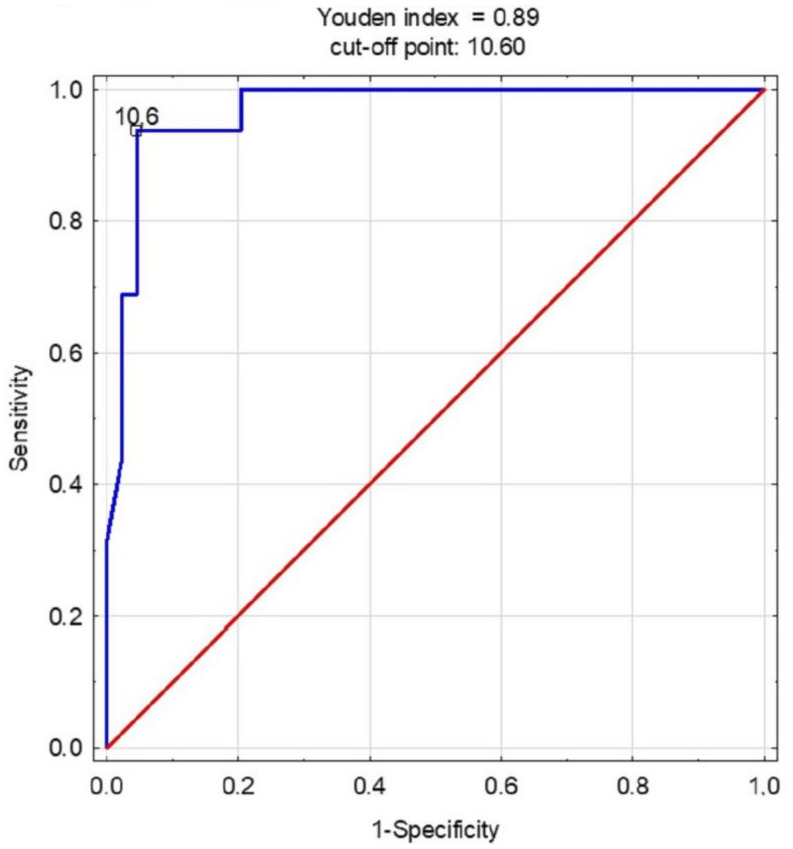
The receiver operating characteristic curve for the SSNDT in all players.

Figure 1 presents the ROC curve for all the players. The SSNDT excellently predicted the risk of injuries in relation to the navicular drop between the static and weighted positions. With an AUC = 97 (95%CI: 0.93–1.00) and *p* < 0.000, the prediction threshold was calculated as a 10.6 mm drop.

**Figure 2:**
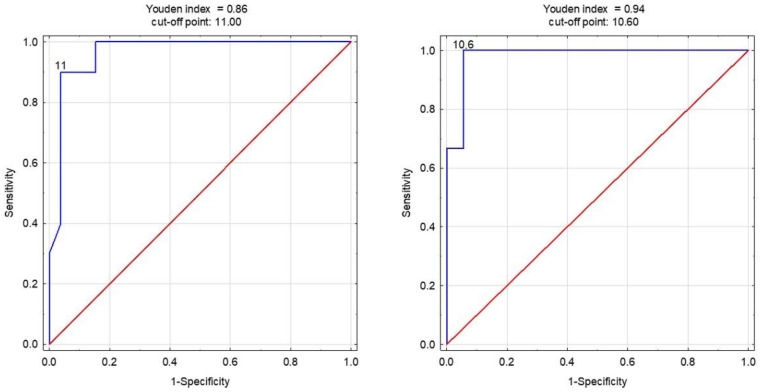
The receiver operating characteristic curves for the SSNDT in academic- (**left**) and top-level (**right**) players.

The diagnostic accuracy of the cut-off points for predicting injuries using the SSNDT in the equation was higher than what would be expected by chance (AUC > 0.5) for both groups of players (Table 4). The SSNDT was an excellent predictor, and its utility in detecting injuries was not limited. The statistics for the academic players were as follows: AUC = 0.963, *p* < 0.001, and cut-off point = 11 mm, while in the top-level players, the AUC was 0.981, *p* < 0.001, and the cut-off point was 10.6. Comparing the AUC between both groups showed no statistically significant differences (difference = −0.018, SE = 0.04, z = −0.47, *p* = 0.627). The ROC curves are presented in Figure 2.

The last part of the analysis involved building simple and multivariate prediction models (using logistic regressions), with a lack of injury also being modeled. For the academic-level players, the lower the SSNDT, the lower the chance of foot injury (β_1_ = −1.23). The odds ratios showed that players with a neutral foot had a 70% less chance of injury than those with a pronated foot. In the top-level players, the observations were similar (β_1_ = −2.71), with the odds ratio showing that the chance of injury was lower (over 90%) in players with a neutral foot than those with a pronated foot. However, the predictors in the top-level players were not statistically significant (Table 5), perhaps due to the low number of participants.

The multivariate logistic regression model confirmed a decreasing chance of injury with a lower SSNDT (β_1_ = −1.44). However, there was no significant influence of competition level on the chance of injury (Table 5).

## 4. Discussion

The current research aimed to (1) assess the prevalence of lower limb injuries in two groups of male players from different competitive levels in relation to foot mobility, (2) verify the potential of the navicular drop (testing the mobility of the foot) in predicting the risk of injuries, and (3) investigate the probability of injury (the chance) in relation to foot mobility and competitive level. The usefulness of the static-to-stance NDT was assessed independently by an examiner well-trained in anatomy by evaluating the reliability of within-day measurements, which demonstrated good reliability for determining foot structure and mobility.

The findings of this work suggest similar injury prevalence in academic- and top-level players since there was a relationship between the pronated foot and risk of injury independent of competitive level. Furthermore, a navicular drop of over 10 mm was a very good predictor of injury in both groups. The risk of injury, if the foot was pronated, ranged from 70% (academic) to over 90% (top-level players). However, no statistically significant effect of the competitive level on the chance of injury was observed.

The rater reliability was good, suggesting the usefulness of the NDT in assessing foot posture. Although the model of analysis (ICC (3.1)) does not allow for generalization of the results to the population, it indicates the high value of the functional foot test. Vauhnik et al. [29] showed that the ICCs for the dominant and non-dominant legs were 0.78 and 0.88, respectively, indicating a moderate to good level of intra-rater reliability when using the NDT to assess foot pronation in clinical settings. However, the results suggest that the NDT can be reliably used in clinical settings for diagnostic and preventive assessment of the foot and ankle. Despite this, caution is needed if the test is used as an evaluation tool in a research setting since it is essential to attain a higher level of reliability before considering its use for research purposes.

The results provided by Deng et al. [26] showed that NH when standing or sitting demonstrated good intra- and inter-rater reliability (ICC = 0.83 to 0.95). The SSNDT had moderate intra- (ICC = 0.68 to 0.78) and inter-rater reliability (ICC = 0.72). Additionally, they showed that the SSNDT lacked predictive validity for gait analysis, and there was no correlation between the SSNDT and dynamic navicular motion in gait (DND), suggesting static NH changes may not predict dynamic alterations. Also, Park et al. [37] demonstrated that the intra-rater reliability of navicular drop measurements ranged from 0.93 to 0.87, while the inter-rater reliability varied from 0.98 to 0.70 when the patient was in both standing and sitting positions. These findings indicate good reliability for the calculated variables. Notably, the intra-rater and inter-rater reliability of the navicular drop in the standing position surpassed that in the sitting position.

The prevalence of injuries in top-level and academic-level volleyball players was very similar, and there were no significant differences in injured and non-injured fractions of the players at different competitive levels. A study by Zhang et al. determined the prevalence of chronic ankle instability (CAI) among elite athletes, finding that 85.9% (170/198) had experienced prior ankle sprains, with a CAI prevalence of 64.6% (128/198). Notably, female athletes exhibited a significantly higher CAI prevalence than their male counterparts. CAI prevention measures are a priority, especially for acrobatic athletes who face an elevated risk. When implementing ankle protection strategies, considering gender and sports categories becomes crucial [38].

Volleyball exhibits a generally low occurrence of injuries, particularly severe ones [19]. The recent literature aligns with past research indicating that ankle sprains remain the most prevalent injuries, especially during competitions. Knee injuries continue to be most prevalent in overuse injuries, while finger injuries, mostly during blocking, remain significant. Data from the NCAA ISS suggest a declining trend in injury rates, potentially attributed to volleyball-focused injury prevention research. Indeed, programs targeting ankle injuries have demonstrated efficacy, with ankle braces showing potential for players without prior ankle injuries [19]. According to previous research [19], despite ample reporting at the collegiate level, injury rates in professional volleyball lack robustness and consistent data. The existing studies on professional athletes are limited to tournament play and omit information on concussions.

The performance of the NDT in predicting the risk of lower limb injury was excellent in both groups of players, although it is difficult to compare our results with those of others due to the lack of such analyses. Using the ROC curve method proved to be a valuable approach to injury risk identification [39], with thresholds of 11 for academic and 10.6 for professional senior players, indicating a higher risk of injury when the foot shows a pronated position, which was similar in both groups. Bere et al. [17] confirmed that the risk of injuries is minimal among elite volleyball players, though senior athletes face a greater susceptibility to injury than their junior counterparts.

Injury risk and patterns are contingent upon player roles. Therefore, preventive strategies should prioritize addressing acute ankle and finger sprains and overuse injuries in the knee, lower back, and shoulder. However, the identification of injury factors is crucial. Kela et al. [40] reported a higher incidence of injuries among male participants than their female counterparts in the same injury category. Additionally, injuries predominantly occurred in body positions such as shoulders, wrists, and ankles for most volleyball players, accounting for 20–63% of injuries in a single season. One of the factors associated with injury risk is foot state, although previous results have shown limited evidence that a pronated foot posture is associated with an increased risk of medial tibial stress syndrome (MTSS) and patellofemoral pain [41]. However, this connection has a small effect, suggesting that the pronated foot posture may contribute to the overall injury risk for these conditions. It is important to interpret these findings cautiously due to the limited evidence available. Among the measures utilized in current prospective research, the navicular drop and Foot Posture Index (FPI) seem to predict lower limb overuse injuries, but dynamic foot function measures may have stronger correlations with injury risk. As such, static foot posture assessments should be incorporated into a comprehensive injury risk assessment and not evaluated in isolation [41].

We are aware that this study had some limitations, including the sample size, using only male players, and its retrospective design. As such, future studies should involve more players of various levels and females, while a prospective study would verify the prognostic potential of the methods used. In addition, it would be interesting to study the impact of the injury as a third variable effect, e.g., a mediating role in the relationships among the physical performance components [42].

## 5. Conclusions

Our study found that the prevalence of foot injuries was rather high among volleyball players (ranging from 25% at the top level to 27.8% at the academic level). Moreover, the number of injured players dramatically increased if the foot was pronated. Thus, the relationship between pronation of the foot and injury is reflected in an increased risk. Top-level players had a significantly higher risk of foot injuries than academic athletes, suggesting that there is a need for specifically tailored prevention. However, when applying foot protection, the competitive level should be considered. In-depth analyses of the mechanisms in the background of volleyball injuries, in relation to competitive level, are still required. Also, there is a need for further consideration of injury risk for specific areas, such as the hip, knee, ankle, and foot, which would enhance preventive strategies.

The practical findings are related to the reliability of the SSNDT, which can be used to assess the risk of musculoskeletal injuries of the foot. It is a simple, fast, low-cost method that can be applied at every competitive level. The measurements conducted by well-prepared anatomically oriented staff were reliable. Secondly, players with a shift of more than 10 mm should be conscious of the potential risk of a foot injury, and this is independent of the competition level. Our results showed that practitioners should also pay attention to the feet during musculoskeletal system screening and introduce pre-rehabilitation procedures aimed at injury prevention when needed.

## Figures and Tables

**Table 1:** Descriptive statistics of the anthropometric measurements and results for the SSNDT.

Measurement	Mean	95%CI		SD
Age [years]	21.1	20.2	22.0	1.2
Body height [cm]	182.5	177.8	187.3	6.2
Body mass [kg]	73.5	68.5	78.5	6.5
BMI [kg/m^2^]	22.1	20.3	23.9	2.3
Foot length—right [cm]	26.1	25.1	27.0	1.2
Foot length—left [cm]	25.6	24.7	26.5	1.2
SSNDT 1st [mm]	9.5	8.3	10.7	2.4
SSNDT 2nd [mm]	9.1	7.7	10.4	2.7

Abbreviations: BMI—Body Mass Index; SSNDT 1st—first sit-to-stand navicular drop test of the day; SSNDT 2nd—second sit-to-stand navicular drop test of the day.

**Table 2:** Descriptive statistics of the anthropometrical measurements and results for the SSNDT.

Measurement	Whole Group				Academic Level				Top Level				t	*p*
	Mean	95%CI		SD	Mean	95%CI		SD	Mean	95%CI		SD		
Age [y]	21.0	20.1	21.9	2.4	20.1	19.6	20.6	1.0	22.5	20.5	24.5	3.2	−3.05	0.005
Body height [cm]	185.8	182.9	188.6	7.6	184.0	180.4	187.5	7.1	188.4	183.4	193.4	7.8	−1.60	0.120
Body mass [kg]	81.4	76.7	86.0	12.3	79.4	72.8	86.1	13.3	84.2	77.5	90.9	10.6	−1.04	0.307
BMI [kg/m^2^]	23.5	22.6	24.4	2.5	23.4	22.0	24.7	2.7	23.7	22.3	25.0	2.1	−0.33	0.744
Foot length—right [cm]	27.0	26.2	27.7	1.9	26.7	25.7	27.6	1.9	27.3	26.1	28.5	1.9	−0.91	0.372
Foot lengt—left [cm]	27.1	26.4	27.8	1.9	26.6	25.7	27.5	1.8	27.8	26.6	28.9	1.8	−1.70	0.100
SSNDT [mm]	8.5	7.8	9.3	2.9	8.6	4.9	15.3	2.8	8.6	4.6	13.5	2.6	0.04	0.973

Abbreviations: SSNDT—sit-to-stand navicular drop test; BMI—body mass index.

**Table 3:** Numbers, percentages, and chi-squared test results for differences in proportions between injured and non-injured feet (separately for both players groups) and categories of the SSNDT results (neutral and pronated foot).

Group	Academic Level	Top Level	Whole Group		Academic Level		Top Level	
			Neutral	Pronated	Neutral	Pronated	Neutral	Pronated
Injured	10 (27.8%)	6 (25%)	1 (2.5%)	15 (75%)	1 (3.85%)	9 (90%)	0 (0%)	6 (60%)
Non-injured	26 (72.2%)	18 (75%)	39 (97.5%)	5 (25%)	25 (96.15)	1 (10%)	14 (100%)	4 (40%)
statistics	χ^2^ = 0.06. *p* = 0.811 φ = −0.03		χ^2^ = 37.7. *p* < 0.001 φ = 0.77		χ^2^ = 27.6. *p* < 0.001 φ = 0.86		χ^2^ = 11.2. *p* < 0.001 φ = 0.68	

**Table 4:** Area under the curve (AUC) and respective cut-off points (Youden) across the SSNDT values.

Group	Cut-Off Point	Youden Index	AUC	SE	95% CI		*p*
Whole	10.6	0.89	0.969	0.02	0.929	1	23.056
Academic level	11.0	0.86	0.963	0.029	0.906	1	15.733
Top-level	10.6	0.94	0.981	0.023	0.936	1	20.603

**Table 5:** Results from simple and multivariate logistic regression models containing the SSNDT for the right and left foot (in simple models) and competitive levels (full, multivariate model).

Group	Term	β Estimate	SE	95%CI		*p*-Value	OR	95%CI	
Academic level	Intercept β_0_	12.66	4.03	4.76	20.56	0.00			
	SSNDT β_1_	−1.23	0.41	−2.03	−0.44	0.00	0.29	0.13	0.64
Top-level	Intercept β_0_	29.28	18.22	−6.43	64.99	0.11			
	SSNDT β_1_	−2.71	1.69	−6.03	0.62	0.11	0.07	0.00	1.85
Whole	Intercept β_0_	15.21	4.24	6.91	23.52	0.00			
	SSNDT β_1_	−1.44	0.41	−2.24	−0.64	0.00	0.24	0.11	0.52
	Competitive level β_2_	−0.50	0.58	−1.63	0.64	0.39	0.37	0.04	3.57

Abbreviations: SSNDT—sit-to-stand navicular drop test.

## Data Availability

The data presented in this study are available upon request from the corresponding author.
